# In Vitro Mouse Ovarian Follicle Growth and Maturation in Alginate Hydrogel: Current State of the Art

**Published:** 2015

**Authors:** M. A. Filatov, Y. V. Khramova, M. L. Semenova

**Affiliations:** Faculty of Biology, Lomonosov Moscow State University, Leninskie Gory, 1, bld. 12, Moscow, 119991, Russia

**Keywords:** alginate, hydrogel, follicle, ovary, mice

## Abstract

This review describes the main factors affecting the *in vitro *development of mouse ovarian follicles under conditions of three-dimensional alginate hydrogel system. The factors discussed include concentration of alginate hydrogel, presence of additives (collagen, fibrin) influencing substrate rigidity; culture conditions; composition of culture media; substances that act like antioxidants (salts of ascorbic acid, glutathione) and contribute to the improvement of lipid metabolism (*L*-carnitine), hormones and growth factors. The methods for follicle group cultivation in alginate hydrogel and cocultivation of different cell populations with follicles encapsulated in alginate hydrogel are covered in the present article.

## INTRODUCTION

Researchers show great interest in designing a system that would allow *in vitro *producing mature oocytes. For various reasons, a woman may require mature oocytes produced *in vitro *from ovarian tissue. For example, ovariectomy is used in hormone-dependent breast cancer to reduce secretion of sex hormones [1]. Radio- and chemotherapy are used to treat other cancer types and also have a negative effect on ovarian status. Chemotherapeutic agents make ovarian cells degrade via the apoptotic pathway. Both the stroma and the follicular ovarian systems are involved in this process [2] by decreasing the number of primordial follicles, reducing the ovarian reserve and causing infertility [3]. Ovarian tissue cryopreservation is currently offered to patients with an eye to returning this tissue to the organism after successful treatment [4]. This procedure is associated with a number of challenges, since both harvesting the ovarian tissue and autografting require surgical intervention and hormonal stimulation to initiate follicle growth and maturation. All these manipulations may have a negative effect on the debilitated condition of the female patient, including increasing the risk of cancer recurrence.

*In vitro *culturing of ovarian tissue followed by production of mature fertilizable oocytes that can be subjected to cryopreservation and subsequently used in assisted reproductive technology programs is a way out of situations such us this one. The methods for *in vitro *culturing of ovarian tissue and individual follicles are currently being actively developed using various animal models, including dog [5] and rhesus macaque [6] models; however, mouse ovarian tissue is used most commonly [7-11]. Although encouraging results have recently been obtained [7, 8, 10], many questions regarding the regulation of follicle growth and oocyte maturation under *in vitro *conditions are far from being solved. Regulation of follicle growth and oocyte maturation* in vivo *depends on hormones secreted by the pituitary gland and ovarian cells, growth factors, as well as other substances whose role remains to be elucidated. Furthermore, follicle growth largely depends on the mechanical properties of the surrounding ovarian tissue. All these conditions need to be provided to successfully culture follicles *in vitro*.

Many researchers culture individual follicles isolated from the ovary either mechanically or enzymatically. A larger number of follicular cells (including theca cells) are preserved when the mechanical method is used, which contributes to better follicle growth *in vitro *[12, 13].

Individual follicles can be cultured in 2D (planar, two-dimensional) or 3D (spatial, three-dimensional) systems. 3D systems have a number of advantages over 2D ones. The main drawback of 2D systems is that the microenvironment of the cultured fragment shows poor correlation to the *in vivo *conditions. In two-dimensional culturing, cells of the follicle and the surrounding stroma migrate within the plane, follicles lose their shape, and oocytes are deprived of the normal cellular environment. Functioning of 2D systems can be regulated only by varying concentrations of chemical agents (growth factors, hormones, etc.) in the culture medium, while 3D culturing technologies allow one to perform regulation by selecting the optimal physical parameters of 3D microenvironment of the explant.

**Fig. 1:**
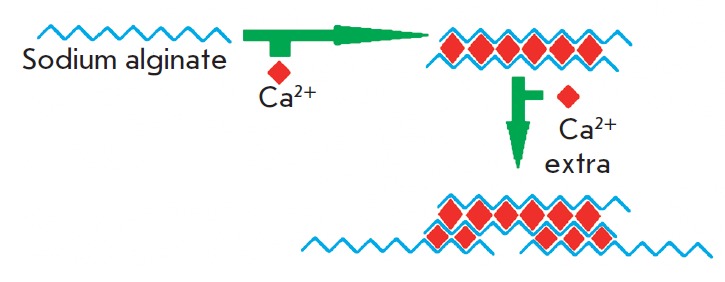
Scheme of alginate hydrogel formation

Agarose, collagen, matrigel, or alginate derivatives are used as substrates when designing 3D culture systems [14]. Alginate hydrogels resulting from dissolution of alginic acid salts (alginates) are advantageous over other substances: it is sufficient to add a solution containing the binding agent (Ca^2+^ or Mg^2+^ ions) to induce their polymerization. Exposure to neither high temperatures nor UV radiation fatal to living cells is needed. Furthermore, alginates are of vegetative origin (they are derived from brown algae), so they can be employed in projects with the limitation to use only systems with animal-free components. *Fig. 1* shows the scheme of formation of alginate hydrogel due to polymerization of sodium alginate induced by calcium ions. Hydrogels having different structures and densities and thus exhibiting different mechanical properties can be produced by varying concentrations of calcium and magnesium salts used in polymerization, alginate solution concentration, and polymerization duration.

**Mechanical environment surrounding the follicle**

Mechanical strains emerging in follicular cells play a crucial role in normal follicle growth and oocyte maturation. Studies by several independent groups of researchers focused on gene expression in mouse ovarian follicles have demonstrated that cultivation of follicles in less concentrated, and therefore softer, alginate substrates better simulates the *in vivo *conditions of follicle growth and maturation than cultivation in more rigid alginate substrates [9, 15, 16].

The number of transcripts of the *Gdf9*, *Bmp15*, *Tcl1* and *Zp3 *genes in oocytes increased as follicles were cultivated in the presence of 0.25% alginate hydrogels (0.25 g sodium alginate dissolved in 100 mL of the solution) as compared to 1.5% alginate hydrogel (1.5 g sodium alginate dissolved in 100 mL of the solution). The high expression level of these genes is typical of normal *in vivo *oogenesis. Furthermore, cultivation of follicles in softer alginate hydrogels (0.25%) significantly increased oocyte and follicle size compared to cultivation in more rigid alginate hydrogel (1.5%) [9].

A group of U.S. researchers have demonstrated that cultivation of follicles in rigid alginate hydrogels (1.5%) that reduce the follicle growth and development rates compared to cultivation in softer alginate hydrogels (0.5%) results in excessive expression of such genes as *Star *(regulating the extracellular transport of cholesterol required for sex hormone synthesis),* Cyp11a1 *(responsible for conversion of cholesterol to pregnenolone), and *Hsd3b1 *(encoding hydroxy-δ- 5-steroid dehydrogenase). Moreover, the expression level of the *Lhcgr *gene, the gene encoding luteinizing hormone and chorionic gonadotropin, was enhanced by cultivation in 0.5% alginate hydrogel as compared to 1.5% hydrogel. The expression level of the *Cyp19a1* gene responsible for conversion of androstendione to estradiol also increased between day 0 and day 8 of follicle cultivation in granulosa cells. This process was more efficient in 0.5% alginate hydrogel: the *Cyp19a1* expression level increased 34-fold, cultivation in 1.5% hydrogel resulted in an only 15-fold rise. As a result, a considerably lower level of estradiol secretion was observed in follicles cultured using the more rigid substrates [15].

It is also of special interest to compare the levels of gene expression for ovarian follicles developed *in vivo* and cultured *in vitro *in alginate hydrogel [16]. Follicles containing two granulosa cell layers (double-layered follicles) were isolated from the ovaries of 12-day-old sexually immature mice, encapsulated in 0.25% alginate hydrogel and cultured for 4 days. The gene expression levels in follicular cells after cultivation in alginate hydrogel and in multilayered follicular cells isolated from the ovaries of 16-day-old mice were compared. The isolated follicles were 150–180 µm in diameter and corresponded to the size of follicles cultured *in vitro*. It was found that ovarian follicles cultured in 0.25% alginate hydrogel and those developing *in vivo *had similar expression patterns, including expression of such genes as *Fshr *(encoding FSH receptor), *Inha *(responsible for the formation of inhibin α-subunit), *Igf1 *(insulin-like growth factor 1 having an effect on follicle growth),* Zp2 *(encoding one of zona pellucida glycoproteins), and* Lhcgr*.

Although soft alginate hydrogels provide better results for follicle cultivation than the more rigid microenvironment, cultivation in hydrogels with a low alginate concentration is associated with a number of technological challenges. Destruction of hydrogel drops occurs quicker in less concentrated solutions, since fewer cross-links are formed between alginate molecules and the structure is weaker. Alginate solutions with a low concentration show promise for cultivation technologies where follicle is encapsulated into the hydrogel and the culture medium is replaced in such a way that hydrogel is not damaged.

***In vitro* reconstruction of the corticalmedullary structure of the ovary**

During *in vivo *growth, a follicle migrates from the rigid area of the ovary (cortex) to the less rigid area (medulla) [17]. Thus, mechanical strains are gradually reduced in follicular cells under natural conditions. Two different methods that use alginates and allow one to reconstruct the cortical-medullary structure for an individual follicle have been designed: cultivation in fibrin–alginate or alginate–collagen hydrogel.

In order to simulate the conditions of variable mechanical strains [17-20], methods for culturing follicles in fibrin–alginate hydrogel were developed; this hydrogel is formed by simultaneous polymerization of alginate and fibrin (alginate polymerization is induced by calcium ions (Ca^2+^), while fibrin polymerization is induced by thrombin and blood coagulation factor XIII). A growing follicle releases lytic enzymes (proteases) that destroy the polymerized fibrin. The mechanical strains existing around the follicle being cultured in fibrin–lginate hydrogel are reduced, resulting in a further increase in follicle size [18]. Fibrin–lginate hydrogel based on 0.25% alginate significantly enhances estradiol and progesterone secretion by follicles and oocyte size increases to a much greater extent than when the standard 0.25% alginate hydrogel without fibrin additive is used [20].

**Fig. 2:**
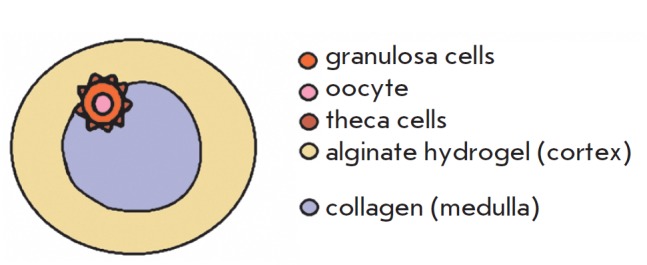
Scheme of alginate–collagen system structure. Follicle is located in the transient zone of mechanical forces between the cortex (the more rigid region) and medulla (softer region). During development follicle transits into the medulla

The alginate–collagen system is another way to provide a dynamic environment of the follicle characterized by different rigidity levels. This type of cultivation suggests that collagen is located in the center of a drop with a follicle encapsulated in it, while alginate hydrogel is present at the periphery. Collagen is a much softer substrate compared to alginate hydrogel; hence, the resulting system is heterogeneous in terms of its rigidity and imitates the follicular microenvironment in the ovary under *in vivo *conditions: the cortex is more rigid, while the medulla is softer [21]. *Fig. 2* shows the scheme of alginate–collagen drop structure with an encapsulated follicle.

The method for follicle encapsulation in the double- layered alginate–collagen system using the microfluidics technology has recently been developed. This technology makes it possible to produce drops consisting of different substances taken at desired ratios due to the directed microflows of different fluids that are generated in a pre-made chip according to a particular scheme [22]. This technology can be used to produce drops of desired size, including those corresponding to the volume of an individual follicle, which simplifies the further cultivation stages [21].

**Table 1:** Development of the procedures for cultivation of mouse ovarian follicles in alginate hydrogels of different compositions (according to [23] with modifications)

Hydrogel composition	Cultivation duration, days	Initial follicle size, µm	Survival rate, %	MII phase reached, %	Additional observations	Source
2% alginate (cortex), 0.5% alginate (core) vs 2% alginate (cortex), 0.5% type I collagen (core)	More than 9	100–130	No data available	No data available	The antral follicle stage is reached more often when collagen is used	[21]
Alginate, 0.25% vs 1.5%	8	130–150	No data available	86 vs 63.8	The expression levels of the main folliculogenesis genes are higher for softer substrates	[9]
0.25% alginate–fibrin	8	130–150	No data available	No data available	Follicle destroys fibrin by proteases, thus reducing mechanical strain	[18]
0.25% alginate–fibrin	12	No data available	75	88	Formation of 2-cell embryos after fertilization of oocytes derived from follicles in vitro	[20]
0.25% alginate	12	100-130	78	59	Investigation of gap junctions in follicular cells	[24]
Alginate, 1.5% vs 0.5%	2-8	150–180	82.7 vs 84.3	No data available	Soft substrate (0.5%) facilitates follicle growth as compared to the more rigid substrate (1.5%)	[15]
0.25% alginate–fibrin	12	100–130	77-81	75-82	No data available	[19]
Alginate, 0.7, 1.5, 3%	8-12	100–130 150–180	31–66 46–91	No data available	Investigation of estrogen secretion by follicles	[25]
Different culture systems (both individual alginate hydrogel and its complexes with various peptides): 1.5% alginate solution 1.5% alginate–type I collagen solution 1.5% alginate–fibronectin solution 1.5% solution of alginate with tripeptides (arginine, glycine, aspartic acid) 1.5% alginate–type IV collagen solution 1.5% alginate–laminin solution	8	100–130 vs 150–180	64 vs 69 65 vs 67 70 vs 72 72 vs 62 72 vs 48 63 vs 61	40 44 71 65 50 71	Cultivation in complexes of alginate with type I collagen and tripeptides resulted in follicle growth; the use of hydrogels containing fibronectin, tripeptides or laminin stimulated formation of oocytes at the MII phase	[26]
Alginate 1.5%	8	150–180	93	71	Birth of live pups after oocytes derived in vitro from follicles were fertilized	[27]
Alginate, 0.25, 0.5, 1, 1.5%	12	100–130	74–85	56–67	Investigation of the effects of substrate rigidity: softer substrates contribute to oocyte development	[28]

*Table 1* lists the main types of follicle culture systems and parameters characterizing the efficiency of these systems, such as the survival rate and the percent of oocytes that reached metaphase II (MII). The best results were achieved by using follicles with an initial diameter of more than 130 µm; follicles less than 100 µm in diameter are not used in these studies, although the number of follicles of this size in the ovary is sufficiently large. A certain minimal volume of cellular microenvironment seems to be needed for successful follicle growth and oocyte maturation in alginate hydrogels.

**Composition of follicle culture media**

A two-stage culture system is usually used to culture follicles encapsulated in alginate hydrogel. At the first stage, cultivation is carried out in the medium facilitating follicle growth, IVC (*In vitro culture medium*). At the second stage, follicles are placed into the *in vitro* maturation medium (IVM). *Table 2* shows the main types of media used to culture follicles encapsulated inside alginate hydrogels and their derivatives.

**Table 2:** Comparison of the media used to culture mouse follicles in alginate hydrogel

Composition of follicle growth medium	Composition of oocyte maturation medium	MII phase reached, %	Source
αMEM, 3 mg/mL BSA, 1 mg/mL fetuin, 10 mIU/mL FSH, 5 µg/mL insulin, 5 µg/mL transferrin, and 5 ng/mL selenite	Not used	No data available	[11]
αMEM, 3 mg/mL BSA, 1 mg/mL bovine fetuin, 5 µg/mL insulin, 5 µg/mL transferrin, ng/mL selenite (ITS), 0.01 IU/mL recombinant human FSH, 50 µg/mL sodium ascorbate	Not used	No data available	[29]
αMEM, GlutaMax (3 mM), penicillin and streptomycin (100 IE/mL), 5 mg/mL human serum albumin, insulin (5 µg/mL), transferrin (5 µg/ mL), selenite (5 ng/mL), ascorbic acid (50 µg/mL), FSH (0.01 IE/mL)	Not used	No data available	[10]
αMEM, 5% ETS, 0.01 IU/mL LH, 0.1 IE/mL FSH, 1 mM L-carnitine	αMEM, 10% ETS, 1.5 IU/mL hCG	51	[7]
αMEM, 1% ETS	αMEM, 10% ETS, 1.5 IU/mL hCG, 5 ng/mL EGF	88	[20]
αMEM, 0.01 IU/mL recombinant FSH, 3 mg/mL BSA, 1 mg/mL bovine fetuin, 5 µg/mL insulin, 5 µg/mL transferrin, 5 ng/mL selenite	αMEM, 10% ETS, 1.5 IU/mL hGC, 5 ng/mL EGF	59	[24]
αMEM, 0.01 IU/mL recombinant FSH, 3 mg/mL BSA, 1 mg/mL bovine fetuin, 5 µg/mL insulin, 5 µg/mL transferrin, 5 ng/mL selenite	αMEM, 10% ETS, 1.5 IU/mL hCG, 5 ng/mL EGF	75–82	[19]
αMEM, 0.01 IU/mL recombinant FSH, 3 mg/mL BSA, 1 mg/mL bovine fetuin, 5 µg/mL insulin, 5 µg/mL transferrin, 5 ng/mL selenite	αMEM, 0.25 pg/mL EGF, 0.045 IU/mL hCG	No data available	[25]
αMEM, 0.01 IU/mL recombinant FSH, 3 mg/mL BSA, 5 µg/mL insulin, 5 µg/mL transferrin, 5 ng/mL selenite	αMEM, 1.5 IU/mL hCG, 5 ng/mL EGF	40–71	[29]

α-MEM complete medium supplemented with different additives is typically used for follicle growth and maturation. To stimulate follicle growth, the medium is supplemented with insulin, selenite, transferrin (ITS), bovine serum albumin (BSA) or fetal calf serum (FCS), and follicle-stimulating hormone (FSH). Substances exhibiting antioxidant properties (ascorbic acid) or enhancing lipid metabolism (L-carnitine) are sometimes added to the culture medium.

The oocyte maturation medium is always supplemented with human chorionic gonadotropin (hCG) to stimulate ovulation. Growth factors, including epidermal growth factor (EGF) facilitating normal meiosis, are also added to this medium in most studies [30].

No unified culturing procedure has been developed thus far, although most researchers use the two-stage culturing procedure, which allows one to stimulate follicle growth and subsequently induce oocyte maturation in them. The first-priority aim of further studies focused on *in vitro *production of fertilizable oocytes is to investigate whether it is reasonable to supplement the two-stage culture systems with various additives capable of inducing follicle and oocyte growth and development or not.

**Regulation of lipid metabolism in folliculogenesis**

Significant attention during *in vitro *follicle culturing is typically given to carbohydrate metabolism. In most cases, carbohydrates are added to the medium as an energy substrate: α-MEM containing sodium pyruvate is the main component of most follicle culture systems. Both the carbohydrate trophic pathway and the lipid β-oxidation pathway are essential for proper oocyte and embryo development. However, only sporadic studies focused on lipid metabolism during follicle and embryo cultivation [7, 31]. Lipid metabolism via the β-oxidation pathway requires carnitine, which facilitates lipid penetration into the mitochondria by being involved in the formation of the so-called carnitine tunnel [32].

ATP is formed during lipid metabolism in mitochondria due to β-oxidation of fatty acids. Fatty acids are activated on the outer surface of the mitochondrial membrane at the first stage of lipid metabolism. ATP, coenzyme A (HS–CoA), and Mg^2+^ ions are involved in activation. The reaction is catalyzed by acyl–CoA synthetase enzyme:

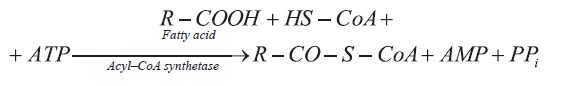



The reaction yields acyl–CoA, which is the active form of the fatty acid. At the second stage of lipid metabolism, the activated fatty acid is supposed to penetrate into the mitochondria. Carnitine palmitoyltransferase I (CPT1B) is the key and simultaneously the limiting factor of this process; this enzyme requires carnitine for functioning [31]. The third stage of lipid metabolism takes place in the mitochondrial matrix, where ATP molecules are synthesized via the citric acid cycle and the electron transport chain [31].

Inhibition of carnitine palmitoyltransferase I was shown to prevent normal meiotic division [31, 33]; hence, a conclusion can be drawn that *L*-carnitine must be used for normal oocyte development and maturation* in vitro*. The experiments involving mouse follicles cultured in alginate substrate [7] demonstrate that supplementation of oocyte maturation medium with* L*-carnitine increases the number of normally developing embryos produced by fertilizing oocytes grown *in vitro *using *L*-carnitine.

Activation of all the metabolic systems (both the carbohydrate and lipid ones) is crucial for stimulating oocyte maturation. Hence, one should expect that there will be further studies focused on the use of *L*-carnitine and other cofactors stimulating lipid metabolism and that new efficient procedures for follicle cultivation giving rise to mature oocytes will be developed.

**Effect of oxidative stress on follicle growth and maturation**

Mammalian ovarian follicles are typically cultured in a conventional carbon dioxide incubator with a 5 vol. % CO_2_/air ratio. Thus, the culturing atmosphere is characterized by the following ratio between the gases (vol. %): 5 CO_2_, 20 O_2_, and 75 N_2_ [34]. At this ratio between the gases in the culturing atmosphere, the partial pressure of oxygen in tissues being cultured is approximately 140 mm Hg [6], while the partial pressure of oxygen in the peritoneal cavity is approximately 40 mm Hg [35], which corresponds to 5 vol. % O_2_ in the atmosphere inside the culture incubator.

The viability of follicles cultured at reduced oxygen concentration increases due to the low level of reactive oxygen species (ROS) formed during cultivation. ROS are actively formed when mammalian ovarian follicles are cultured at increased partial pressure of oxygen, thus inducing oxidative stress in cells that has a negative effect on follicle growth and development [36].

It has been demonstrated in a number of studies [10, 37] that cultivation at low oxygen concentrations (5 vol. % compared to 20 vol. % O_2_) in an incubator atmosphere increases the viability and improves growth of mouse ovarian follicles.

Special attention should be paid to oxygen concentration in an incubator atmosphere at early stages of cultivation of follicles (primordial, primary, and early secondary ones). Under *in vivo *conditions, these follicles reside in the ovarian cortex, whose degree of vascularization is much lower than that in the medulla. As a result, early follicles actually exist under extreme hypoxic conditions. Similar conditions need to be reproduced to culture them *in vitro*. Thus, when early secondary mouse follicles 100–120 µm in diameter are cultured in 2.5 vol. % oxygen atmosphere, their growth, survival rate, and production of vascular endothelial growth factor A (VEGFA), lactate, inhibin B, and anti- Mullerian hormone reliably increase as compared to cultivation in an atmosphere containing 20 vol. % O_2_ [11].

On the other hand, it has been demonstrated that cultivation at elevated oxygen concentrations yields higher quality oocytes both during follicle cultivation [38] and when oocyte–cumulus complexes mature *in vitro *[39]. Low oxygen concentration may disturb activity of motor proteins, including dynein and dynactin, regulatory factors responsible for mitotic spindle formation, and proteins regulating the cell cycle in oocytes [38]. Furthermore, reduced oxygen concentration in the culture medium impairs mitochondrial function, which, in turn, reduces ATP production. This very process may result in impaired function of all the groups of proteins mentioned above. Follicle cultivation and maturation at low oxygen levels may also desynchronize nuclear and cytoplasmic maturation of oocytes [38].

The ambiguity of data on the optimal oxygen concentration in a culture atmosphere makes further research relevant, since quality of the embryos developing from oocytes depends on which conditions were selected. Multistage culture systems combining the use of different oxygen concentrations at different culture stages might be developed. The three-stage culture system shows promise. Ultra-low oxygen concentrations should be used at the first stage (when culturing early follicles). At the second stage (when culturing later antral follicles), oxygen concentration in the incubator atmosphere should be increased. Oxygen concentration in the incubator atmosphere should probably be additionally increased at the third stage (when the oocyte–cumulus complexes isolated from the culture mature). However, the number of culturing stages and the percentage of oxygen in the culture medium at each stage have not been determined yet. It may also be possible that better results are achieved not by changing oxygen concentration in the culturing atmosphere stepwise but by gradually increasing it during the entire cultivation procedure.

The level of reactive oxygen species in follicles being cultured can also be reduced in a different way: by supplementing the culture medium with antioxidants. To reduce the level of formation of reactive oxygen species during follicle cultivation, the medium can be supplemented with various antioxidants, such as quercetin [40], ascorbic acid [29, 41], 7,8-dihydroxyflavone [42], and glutathione [29]. Meanwhile, it has been demonstrated that the positive effect of sodium ascorbate on follicle growth is caused by its ability to stimulate the formation of contacts between follicular cells and the extracellular matrix rather than by antioxidant properties of this substance. Cultivation of mouse follicles encapsulated in alginate hydrogel in the presence of glutathione increased neither their survival rate nor growth compared to the follicles cultured in the medium supplemented with sodium ascorbate. The antioxidant activity of both ascorbic acid and glutathione is most likely to have no significant effect on *in vitro *folliculogenesis [29].

**The use of coculture procedures**

Natural conditions of follicle growth and maturation *in vitro *are imitated by supplementing the culture medium with hormones (FSH, hCG, LH), [7, 16, 17], growth factors [20, 25], and other components [7, 10, 29]. Nevertheless,* in vivo *follicle growth conditions cannot be imitated in a laboratory so far. In addition to hormones, follicle’s granulosa and theca cells produce a large amount of growth factors so that a specific area locally enriched in hormones is formed. This area is most favorable for follicle growth and development and oocyte growth in them.

Some researchers use a fundamentally different approach (follicle group cocultivation) to produce a follicle culture medium supplemented with numerous growth factors and hormones. It is expected that ovarian components during cocultivation of several follicles will stimulate mutual growth and development by enriching the environment in paracrine factors secreted at concentrations required to ensure normal growth of follicles. There are currently three main types of coculture: non-contact coculture of a large number of follicles within one drop of alginate hydrogel [8], coculture of follicles with embryonic fibroblasts [29, 43], and coculture of follicles in a conditioned medium [13, 43]. *Table 3* lists the schemes of the most common follicle coculture systems.

**Table 3:** Main follicle coculture s

Cocultivation type	Experimental scheme	Source
1. Non-contact cultivation of a large number of follicles	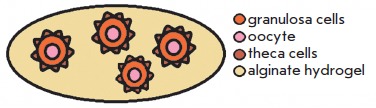	[8]
2. Follicles in the embryonic fibroblasts-conditioned medium	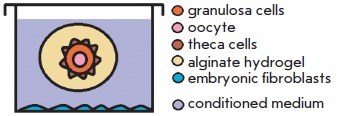	[43]
3. Follicles in the pre-conditioned medium	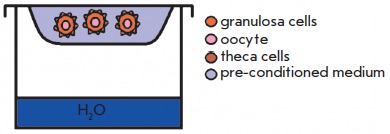	[13]

When performing direct coculture, several follicles are placed in an alginate hydrogel drop; however, follicles do not contact with one another and some space for growth is left between them. Follicles ‘communicate’ by releasing paracrine factors into the environment surrounding the follicles. The best results were achieved by culturing 10 follicles per group [8].

Follicles are cocultured with embryonic fibroblasts as follows: follicles encapsulated in alginate hydrogel are placed onto a monolayer of inactivated mouse embryonic fibroblasts that condition the coculture medium by various paracrine factors. Follicles with smaller diameter (starting with 80–90 µm) can be grown under these conditions more successfully than in feeder layer-free culture systems, which support growth of follicles larger than 100 µm in diameter.

When culturing follicles in a conditioned medium, various cell cultures (mouse embryonic fibroblasts, ovarian cell components) are grown preliminarily; the culture medium is then collected and follicles encapsulated in alginate hydrogel are placed in it. This medium contains various paracrine factors, including the required growth factors that diffuse into the hydrogel, thus forming favorable conditions for follicle growth.

Each of these three main methods has its own advantages and drawbacks. When using non-contact cocultivation of a large number of follicles, it is extremely difficult to control growth and maturation of each individual follicle. Death of a single follicle in the group will also reduce the growth rates of the remaining follicles. Meanwhile, the non-contact cocultivation of follicles is a technologically simple method that allows one to achieve rather good results during cultivation. There are a number of difficulties associated with using the feeder layer of inactivated embryonic fibroblasts to be cocultured with follicles. In particular, the optimal equilibrium between the compositions of culture media needs to be maintained, since the medium needs to contain various substances to ensure normal growth and development of follicles and embryonic fibroblasts. Furthermore, the applicability of this method in medical practice is also an open question, since fetal cells are used during cocultivation in this type of systems. Cultivation of follicles in a pre-conditioned medium is also associated with technological challenges. This coculture system implies several stages, and contamination of the culture medium needs to be avoided at each stage. Furthermore, it is difficult to standardize the process of medium conditioning by proliferating cell population as each batch of the conditioned medium may contain different concentrations of active substances.

## CONCLUSIONS

The technologies for cultivation of mammalian ovarian tissue become more and more advanced and better correlate with *in vivo *conditions. Taking into account the significant number of various aspects, researchers have successfully achieved better cultivation results: the produced oocytes mature in most cases and follicle growth until later stages is observed more often. New components for supplementing culture media to ensure better growth and maturation of follicles are likely to be discovered in future. Further research will also focus on *in vitro *ovarian reconstruction, which will allow one to culture follicles under optimal conditions. A high-performance follicle culture system can be designed with allowance for numerous factors (mechanical strain, follicular metabolism, gas concentration in the culture environment, hormonal background, effect of paracrine factors, etc.). Hence, studies using a combination of all the factors required for normal follicle growth and development can be expected in the near future; these conditions will allow one to produce oocytes characterized by fertilizability and developmental competency.

## Abbreviations

2D: two-dimensional
3D: three-dimensional
FSH: follicle-stimulating hormone
hCG: human chorionic gonadotropin
LH: luteinizing hormone
BSA: bovine serum albumin
FCS: fetal calf serum;
ITS: insulin, transferrin, selenium
EGF: epidermal growth factor
